# miR‐874 directly targets AQP3 to inhibit cell proliferation, mobility and EMT in non‐small cell lung cancer

**DOI:** 10.1111/1759-7714.13428

**Published:** 2020-04-16

**Authors:** Shuhua Wang, Yuanyuan Wu, Shenghua Yang, Xunchao Liu, Yong Lu, Fengxia Liu, Guixia Li, Guirong Tian

**Affiliations:** ^1^ Department of Clinical laboratory Heze Municipal Hospital Heze China; ^2^ Department of Respiratory Medicine Heze Municipal Hospital Heze China; ^3^ Medical Research Laboratory Heze Medical College Heze China; ^4^ Department of Clinical laboratory Juye County Hospital of TCM Heze China; ^5^ Ministry of Science and Education Heze Municipal Hospital Heze China

**Keywords:** AQP3, EMT, miR‐874, non‐small cell lung cancer, PI3K

## Abstract

Non‐small cell lung cancer (NSCLC) is a major type of lung cancer with high morbidity and high mortality. miR‐874 has been determined to play a role in tumor suppression in several cancers. The purpose of our study was to detect the biological mechanisms of miR‐874 and AQP3 in NSCLC.

**Methods:** CCK‐8 and Transwell assays were utilized to perform cell invasion.Western blot was employed to evaluate the protein expression.

**Results:** The expression of miR‐874 was lower in NSCLC tissues than that of corresponding adjacent nontumor tissues. Downregulation of miR‐874 predicted a poor prognosis in NSCLC. The cell proliferation and mobility were suppressed by overexpression of miR‐874, which were promoted by knockdown of miR‐874 in A549 and H1299 cells. miR‐874 mediated the expression of AQP3 by directly binding to the 3′‐untranslated regions (UTR) of AQP3 mRNA in NSCLC cells. Moreover, miR‐874 inhibited the proliferation and mobility by targeting AQP3 and inhibited the PI3K/AKT signaling pathway in A549 cells. miR‐874 inhibited the growth of NSCLC in vivo.

**Conclusions:** In conclusion, miR‐874 inhibited proliferation and mobility by regulating AQP3 in NSCLC. The newly identified miR‐874/AQP3 axis provides novel insight into the pathogenesis of NSCLC.

## Introduction

Lung cancer is the most frequent cancer with the highest morbidity and mortality worldwide, which includes small cell lung cancer and non‐small cell lung cancer (NSCLC).^1^ NSCLC is classified into lung adenocarcinoma, large cell carcinoma and squamous cell carcinoma, with higher mortality.^2^ Therefore, we urgently need to identify new therapeutic targets and molecular mechanisms underlying NSCLC.

MicroRNAs (miRNAs), 19–25 nucleotides long, non‐coding RNA, regulate cell development and progress by directly binding to the 3′‐untranslated regions (UTR) of target mRNAs at post‐transcriptional level.^3^, ^4^, ^5^ miRNAs may act as tumor suppressors or oncogenes to regulate cancer development in NSCLC, including miR‐1258, miR‐1269a, miR‐1179 and miR‐550a.^6^, ^7^, ^8^, ^9^ Previous studies have indicated that miR‐874 is downregulated and acts as a tumor suppressor in hepatocellular carcinoma and melanoma.^10^ In their study, Zhang *et al*. revealed that miR‐874 inhibited the proliferation and metastasis in hepatocellular carcinoma.^11^

Aquaporin 3 (AQP3) with a molecular weight of 30 KDa, is a member of the aquaporin family.^12^, ^13^ AQP3 has been reported to be abnormally expressed in several diseases that include breast, prostate, gastric and pancreatic cancers.^14^, ^15^, ^16^, ^17^ Wang *et al*. elucidated that AQP3 promoted cell proliferation and enhanced cell apoptosis in squamous cell carcinoma.^18^ Additionally, Muhammad *et al*. demonstrated that AQP3 improved cell proliferation, migration, invasion, adherence and response in breast cancer.^19^

In this study, we discovered that miR‐874 exhibited low expression in NSCLC tissues and cell lines and downregulation of miR‐874 predicted poor prognosis of NSCLC patients. miR‐874 inhibited the proliferation and invasion by targeting AQP3 through regulation of the PI3K/AKT signaling pathway and epithelial‐to‐mesenchymal transition (EMT) in NSCLC. What is more, miR‐874 inhibited the growth of NSCLC cells in a mouse xenograft model.

## Methods

### Clinical tissues samples

NSCLC and matched paracancerous tissues were obtained from 49 patients at the Department of Respiratory Medicine, Heze Municipal Hospital from January 2016 to December 2017. Of the 49 patients with NSCLC there were 38 males and 11 females, aged 38–74 years with a mean age of 62.7 years. The fresh tissue samples were immediately frozen in liquid nitrogen and stored at −80°C. The written informed consent for the procedures to be carried out were received from all patients and approved by the Ethical Committee of the Heze Municipal Hospital.

### Cell culture and transfection

Human NSCLC cell lines A549 and H1299 and human normal epithelial cell line BEAS‐2B were obtained from American Type Culture Collection (ATCC; Manassas, VA, USA). All cells were cultured in RPMI‐1640 medium (Gibco‐BRL; Rockville, MD, USA) supplemented with 10% FBS (Thermo Fisher Scientific), which was maintained at 37°C in an atmosphere of 5% CO_2_.

Prior to transfection, A549 cells were seeded in six‐well plates and incubated overnight at 37°C with 5% CO_2_. The A549 cells were transfected with the miR‐874 mimic or the miR‐874 inhibitor (GenePharma, Shanghai, China) were conducted to up‐ or downregulate miR‐874. Moreover, pcDNA3.1‐AQP3 (GenePharma) was employed to overexpress AQP3 in A549 cells using lipofectamine 2000 (Thermo Fisher Scientific).

### RNA isolation and real‐time quantitative PCR (RT‐qPCR)

TRIzol reagent (Thermo Fisher Scientific) was applied to extract the total RNAs from cell lines and tissues. Prime Script RT Reagent Kit (Takara Biotechnology, Dalian, China) and miRNA Reverse Transcription Kit (Life Technologies, Foster, CA, USA) were employed to synthesize the first cDNA chains of mRNA and miRNA, respectively. The SYBR‐Green detection system (Roche Applied Science, Penzberg, Germany) and the MystiCq miRNAs qPCR Assay Primer (Sigma, Missouri, USA) were used to perform the qPCR on Applied Biosystems Step One Plus Real Time PCR System. The expression of miR‐874 and AQP3 were calculated by the 2‐ΔΔCq cycle threshold method,^20^ with U6 and reduced glyceraldehyde‐phosphate dehydrogenase (GAPDH) as normalization respectively. The primers are shown: miR‐874 Forward: 5′‐TGCGGCTGCCCTGGCCCGAGGGAC‐3′, Reverse: 5′‐CCAGTGCAGGGTCCGAGGT‐3′; U6 Forward: 5′‐TGCGGGTGCTCGCTTCGGCAGC‐3′, Reverse: 5′‐CCAGTGCAGGGTCCGAGGT‐3′; AQP3 Forward: 5′‐TCAATGGCTTCTTTGACCAGTTCA‐3′, Reverse: 5′‐CTTCACATGGGCCAGCTTCACATT‐3′; GAPDH Forward: 5′‐GGAGCGAGATCCCTCCAAAAT‐3′, Reverse: 5′‐GGCTGTTGTCATACTTCTCATGG‐3′ (Table 1).

**Table 1:** Primer sequence

Gene	Forward primer 5′‐3′	Reverse primer 5′‐3′
miR‐874	TGCGGCTGCCCTGGCCCGAGGGAC	CCAGTGCAGGGTCCGAGGT
U6	TGCGGGTGCTCGCTTCGGCAGC	CCAGTGCAGGGTCCGAGGT
AQP3	TCAATGGCTTCTTTGACCAGTTCA	CTTCACATGGGCCAGCTTCACATT
GAPDH	GGAGCGAGATCCCTCCAAAAT	GGCTGTTGTCATACTTCTCATGG

### Cell proliferation assay

The cell counting kit‐8 (CCK‐8) assay was employed to measure the proliferation in A549 and H1299 cells. In brief, A549 cells and H1299 cells were seeded in 96‐well plates before transfection. The CCK‐8 solution was added to the medium and cultured for four hours at 37°C, and produced formazan in the bottom of the wells. Finally, the ELISA plate reader (Bio‐Rad Laboratories, Hercules, CA, USA) was conducted to measure the optical density at 570 nm.

### Transwell assay

The invasive capacity was calculated through transwell inserts (Corning Incorporated, Corning, NY, USA), which were coated with Matrigel (BD Biosciences, Franklin Lakes, NJ, USA). We suspended A549 cells in FBS free RPMI‐1640 medium and transferred them into the top of the Matrigel‐coated transwell chambers. Meanwhile, the lower chamber was filled with RPMI‐1640 medium containing 15% FBS as an attractant. After incubation at 37°C overnight, the invading cells in the outside of the membrane were fixed and stained by paraformaldehyde and crystal violet respectively, and the cells were then counted under a microscope (Olympus Corporation, Tokyo, Japan).

### Western blot analysis

An ice cold RIPA lysis buffer (Sigma, USA) containing 10% PMSF (Sigma, USA) was used to lyse the cells for 30 minutes. The protein solving liquid was centrifuged at 12000 × g for 20 minutes at 4°C and the supernatants were collected. Equal amount of proteins were separated through electrophoresis utilizing 10% sodium dodecyl sulfate polyacrylamide gel electrophoresis (SDS‐PAGE) and then transferred to a polyvinylidene fluoride membrane (PVDF; Millipore, USA). After blocking with 5% fat‐free milk in TBST buffer at room temperature for one hour, the blots were then incubated with the primary antibodies at 4°C overnight. The primary antibodies were against AQP3 (1:1000; cat. ab125219, Abcam, Cambridge, USA), E‐cadherin (1:1000; cat. ab1416, Abcam), N‐cadherin (1:1000; cat. ab18203, Abcam), Vimentin (1:1000; cat. ab8978, Abcam), p‐PI3K (1:1000, cat. BS4605, Xinle Biology, Shanghai, China), PI3K (1:1000, cat. MA5‐26514, Thermo Fisher Scientific, Waltham, MA, USA), p‐AKT (1:1000, cat. 4060, Cell Signaling), AKT (1:1000, cat. 4691, cell signaling) and GAPDH. The membranes were subsequently incubated with anti‐rabbit or mouse HRP‐conjugated secondary antibody for two hours at room temperature. Finally, the signals were evaluated by enhanced chemiluminescence (ECL, Pharmacia Biotech, Arlington, USA).

### Luciferase reporter assay

TargetScan predicted that AQP3 had a binding site of miR‐874, and the binding site was located at 351 to 358 on 3′‐UTR of AQP3 mRNA. To validate whether miR‐874 could directly bind to the 3′‐UTR of AQP3 mRNA, the binding sequences were mutated from AGGGC to CACCA and inserted in pmirGLO luciferase reporter vector. The miR‐874 mimic and the wild‐type 3′‐UTR or the mutant 3′‐UTR of AQP3 were cotransfected in A549 cells. The luciferase activity was measured using a dual‐luciferase reporter assay system (Promega) with renilla luciferase activity as the normalization.

### Xenograft tumor formation assay

Nude mice age four weeks old were purchased from Charles River Laboratories (Beijing, China). A549 cells stably transfected with the miR‐874 mimic or negative control were injected subcutaneously. The length and width were measured and recorded every three days, and cultured for 26 days until the mice were executed. All animal experiments were performed in the animal laboratory center of Chengyang People's Hospital and approved by the Animal Care and Use Committee of Chengyang People's Hospital.

### Statistical analysis

The data with normal distribution were expressed as mean ± standard deviation (SD) from at least three independent experiments. The comparisons between two groups and multiple groups were analyzed by Student's *t*‐test and one‐way analysis of variance followed by Turkey multiple comparison post‐hoc analysis. The connections between the expression of miR‐874 and clinicopathological features were analyzed by Chi‐square test and Kaplan‐Meier method. *P* < 0.05 was considered as statistically significant.

## Results

### Downregulation of miR‐874 predicted poor prognosis of NSCLC

**Figure 1:**
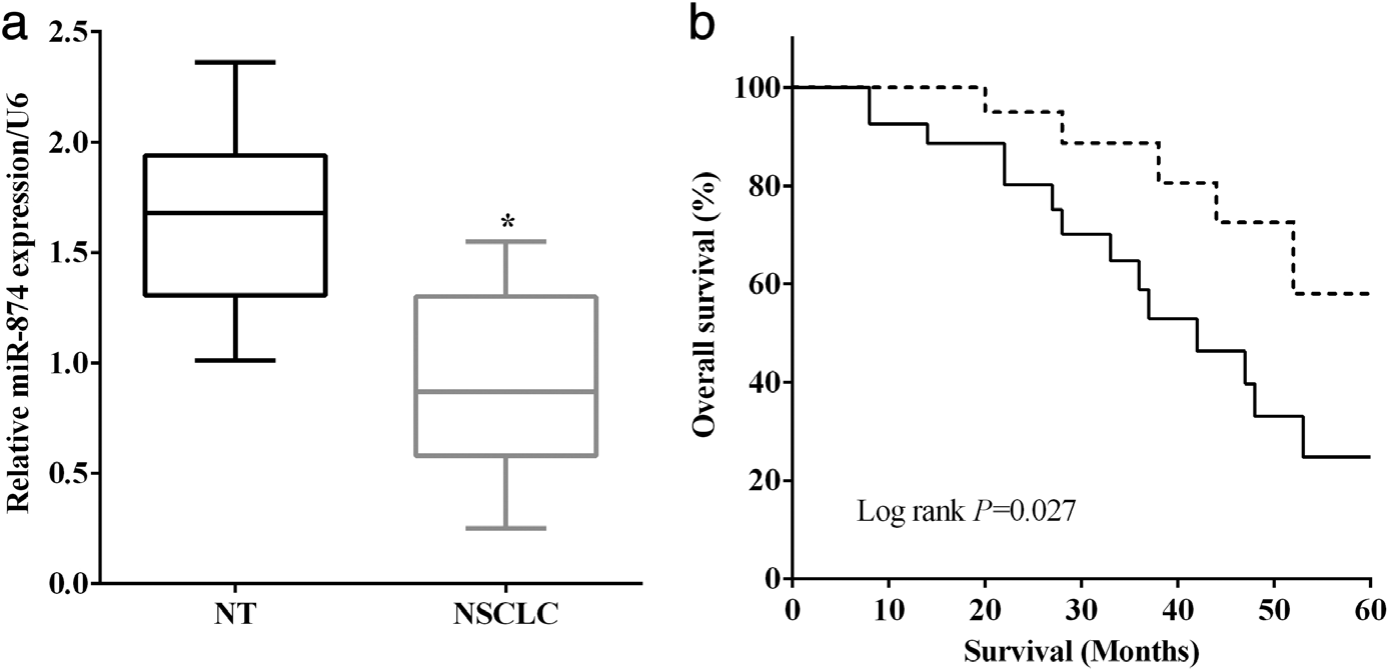
Downregulation of miR‐874 predicted poor prognosis of NSCLC. (**a**) The expression of miR‐874 was lower in NSCLC tissues (*n* = 49) than that of the corresponding nontumor tissues (*n* = 49). (**b**) Log‐rank test of Kaplan‐Meier method revealed that downregulation of miR‐874 predicted poor five‐year survival of NSCLC patients. (**b**) (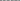) miR‐874(+), (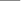) miR‐874.

The miR‐874 levels in 49 pairs of NSCLC and corresponding nontumor tissues were evaluated by RT‐qPCR. As expected, the expression of miR‐874 was lower in NSCLC tissues than that of the corresponding nontumor tissues (*P* < 0.05) (Fig 1a). Moreover, the overall survival was calculated using the log‐rank test of the Kaplan‐Meier method, and we discovered that downregulation of miR‐874 in NSCLC predicted poor five‐year survival (*P* < 0.05) (Fig 1b).

### miR‐874 suppressed the proliferation and mobility in NSCLC cells

**Figure 2:**
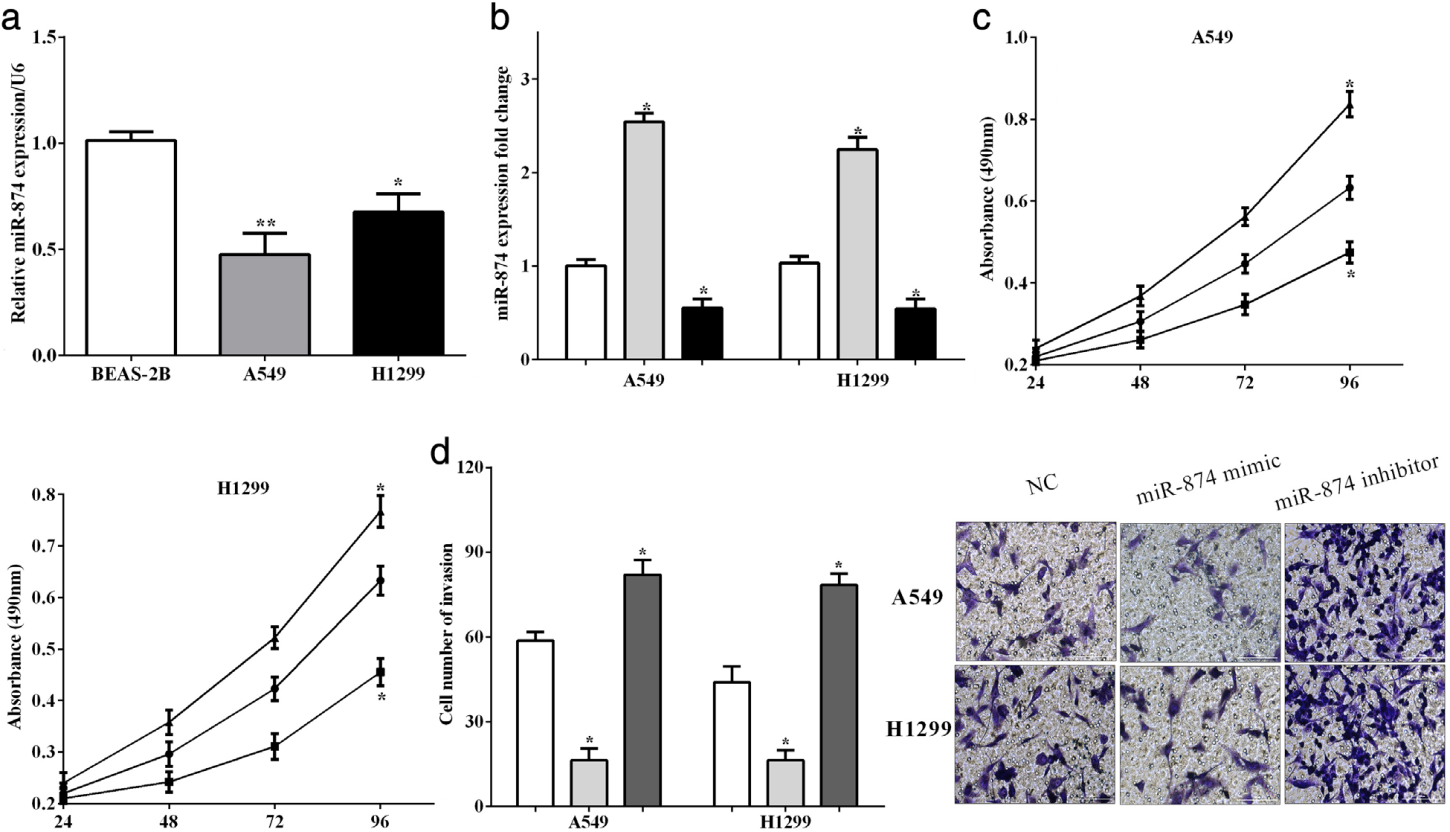
miR‐874 suppressed the proliferation and mobility in NSCLC cells. (**a**) The expression of miR‐874 was higher in BEAS‐2B cells than that of A549 and H1299 cell lines. (**b**) The miR‐874 mimic and the miR‐874 inhibitor were transfected to up‐ or downregulate miR‐874 in A549 cells and H1299 cells. (**c**) Cell counting kit‐8 (CCK‐8) assay indicated the proliferative ability was suppressed by the miR‐874 mimic, while it was promoted by miR‐874 inhibitor in A549 cells and H1299 cells. (**d**) Transwell assay indicated that overexpression of miR‐874 inhibited the mobility ability whereas it was enhanced by miR‐874 inhibitor in A549 cells and H1299 cells. (**a**) (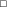) BEAS‐2B, (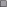) A549, (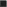) H1299; (**b**) (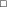) NC, (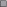) miR‐874 mimic, (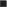) miR‐874 inhibitor; (**c**) (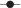) NC, (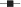) miR‐874 mimic, (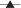) miR‐874 inhibitor; (**d**) (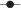) NC, (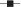) miR‐874 mimic, (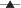) miR‐874 inhibitor; (**e**) (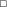) NC, (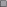) miR‐874 mimic, (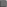) miR‐874 inhibitor.

The expression of miR‐874 were measured in two NSCLC cell lines (A549 and H1299) and a normal bronchial epithelial cell line BEAS‐2B. The expression of miR‐874 was higher in BEAS‐2B cells than NSCLC cell lines A549 (*P* < 0.01) and H1299 (*P* < 0.05) (Fig 2a) which was the same as the findings in tissues. To explore the important functions of miR‐874, the miR‐874 mimic and the miR‐874 inhibitor was transfected to up‐ (*P* < 0.05) or downregulate (*P* < 0.05) miR‐874 in A549 cells and H1299 cells, which were assessed by RT‐qPCR (Fig 2b). CCK‐8 assay indicated the cell proliferative ability was suppressed (*P* < 0.05) by the miR‐874 mimic, while it was increased (*P* < 0.05) by the miR‐874 inhibitor in A549 cells and H1299 cells (Fig 2c). Moreover, transwell assay indicated that cell mobility ability was inhibited by overexpression of miR‐874 (*P* < 0.05) whereas it was enhanced by the miR‐874 inhibitor (*P* < 0.05) in A549 and H1299 cells (Fig 2d). All the results demonstrated that the proliferative and mobility abilities were suppressed by miR‐874 in NSCLC cell lines A549 and H1299.

### miR‐874 regulated expression of AQP3 through directly binding to the 3′‐UTR of AQP3 mRNA

**Figure 3:**
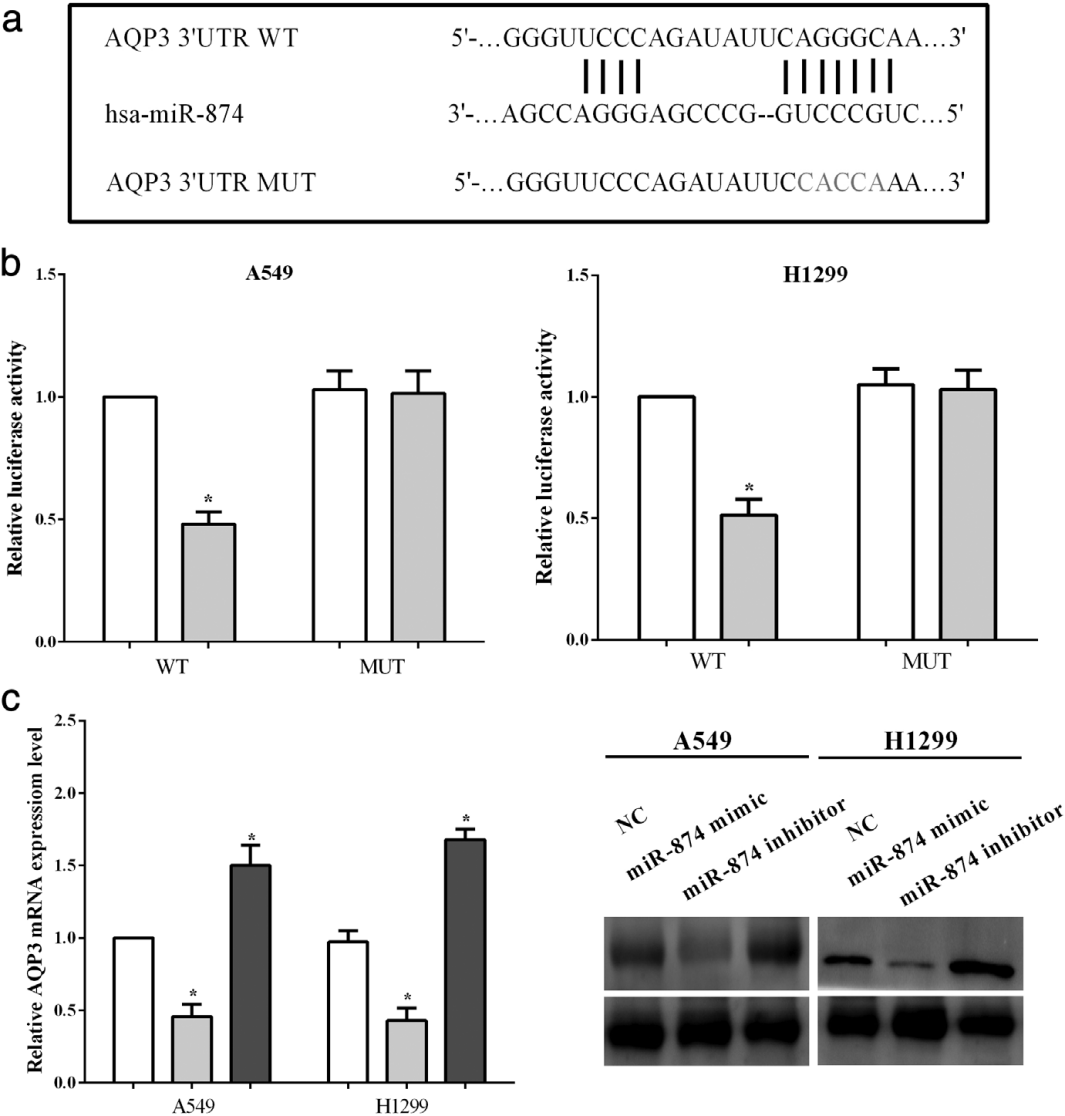
miR‐874 regulated the expression of AQP3 through directly binding to the 3′‐ untranslated regions (UTR) of AQP3 mRNA. (**a**) TargetScan predicted the binding sequences of miR‐874 on 3′‐UTR of AQP3 mRNA. (**b**) The luciferase reporter assay indicated that miR‐874 exhibited direct binding to the 3′‐UTR of AQP3 mRNA. (**c**) The mRNA level of AQP3 was mediated by miR‐874 in A549 cells. (**b**) (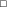) NC, (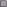) miR‐874 mimic, (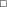) NC, (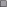) miR‐874 mimic; (**c**) (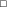) NC, (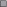) miR‐874 mimic, (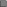) miR‐874 inhibitor.

TargetScan predicted AQP3 was one of the target genes of miR‐874, and the binding site was located at 351–358 on the 3′‐UTR of AQP3 mRNA. The binding sequences of miR‐874 on the 3′‐UTR of AQP3 mRNA were mutated from AGGGC to CACCA, and then the luciferase activity (Fig 3a) was calculated. The luciferase reporter assay indicated that miR‐874 mimic reduced (*P* < 0.05) the luciferase activity of A549 and H1299 cells transfected wild‐type 3′‐UTR of AQP3 mRNA, while it did not alter (*P* < 0.05) the mutant 3′‐UTR of AQP3 mRNA (Fig 3b). Moreover, the mRNA levels of AQP3 were calculated after transfection with the miR‐874 mimic or the miR‐874 inhibitor in A549 cells. As expected, overexpression of miR‐874 suppressed the mRNA level of AQP3 (*P* < 0.05), while knockdown of miR‐874 enhanced the expression of AQP3 in A549 cells and H1299 (*P* < 0.05) (Fig 3c). All the results indicated that miR‐874 regulated the expression of AQP3 through directly binding to the 3′‐UTR of its mRNA in NSCLC cell lines A549 and H1299.

### miR‐874 inhibited EMT and suppressed phosphorylation of PI3K/AKT

**Figure 4:**
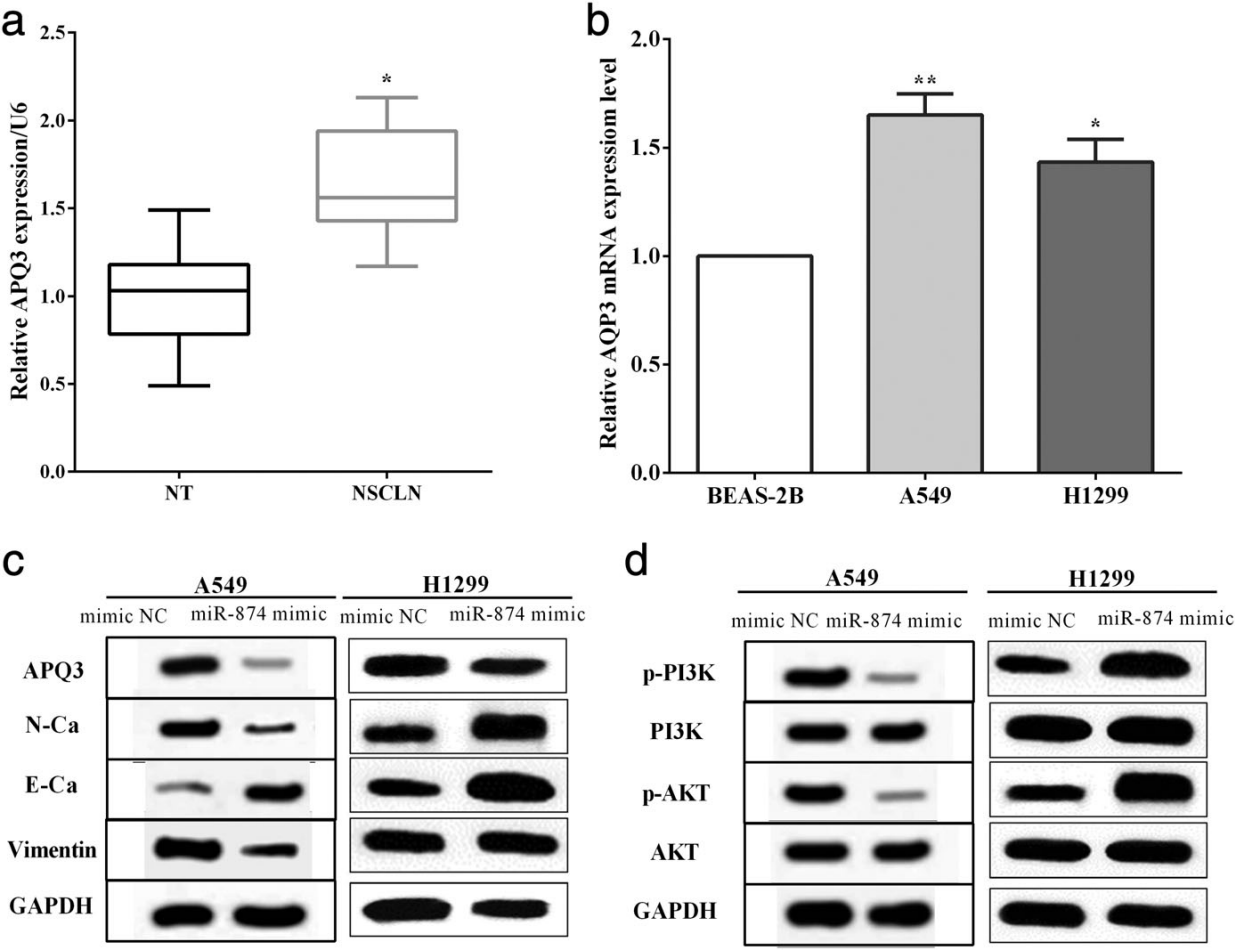
miR‐874 inhibited the EMT and suppressed phosphorylation of PI3K/AKT. (**a**) AQP3 was upregulated in NSCLC tissues (*n* = 49) versus the corresponding nontumor tissues (*n* = 49). (**b**) The expression of AQP3 was higher in A549 and H1299 cells than that of the bronchial epithelial cell line BEAS‐2B. (**c**) The miR‐874 mimic suppressed the EMT through AQP3. (**d**) miR‐874 inhibited phosphorylation of PI3K/AKT in A549 cells. (**b**) (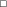) BEAS‐2B, (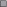) A549, (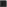) H1299.

qRT‐PCR assay was employed to evaluate the expression of AQP3 in tissues and cell lines, and we discovered that AQP3 was upregulated in NSCLC tissues versus the corresponding nontumor tissues (*P* < 0.05) (Fig 4a). Meanwhile, the expression of AQP3 was higher in A549 (*P* < 0.01) and H1299 (*P* < 0.05) cells than that of bronchial epithelial cell line BEAS‐2B (Fig 4b). In addition, western blot was utilized to assess the protein expression associated with EMT and PI3K pathway in A549 and H1299 cells. We discovered that the miR‐874 mimic suppressed the expression of AQP3 and E‐cadherin, whereas it improved the expression of N‐cadherin and Vimentin in A549 and H1299 cells (Fig 4c), which elucidated that miR‐874 suppressed the EMT through AQP3. Meanwhile, overexpression of miR‐874 inhibited the expression of p‐PI3K and p‐AKT in A549 and H1299 cells (Fig 4d), which revealed that miR‐874 inhibited phosphorylation of PI3K/AKT pathway.

### AQP3 reversed partial functions of miR‐874 in A549 cells

**Figure 5:**
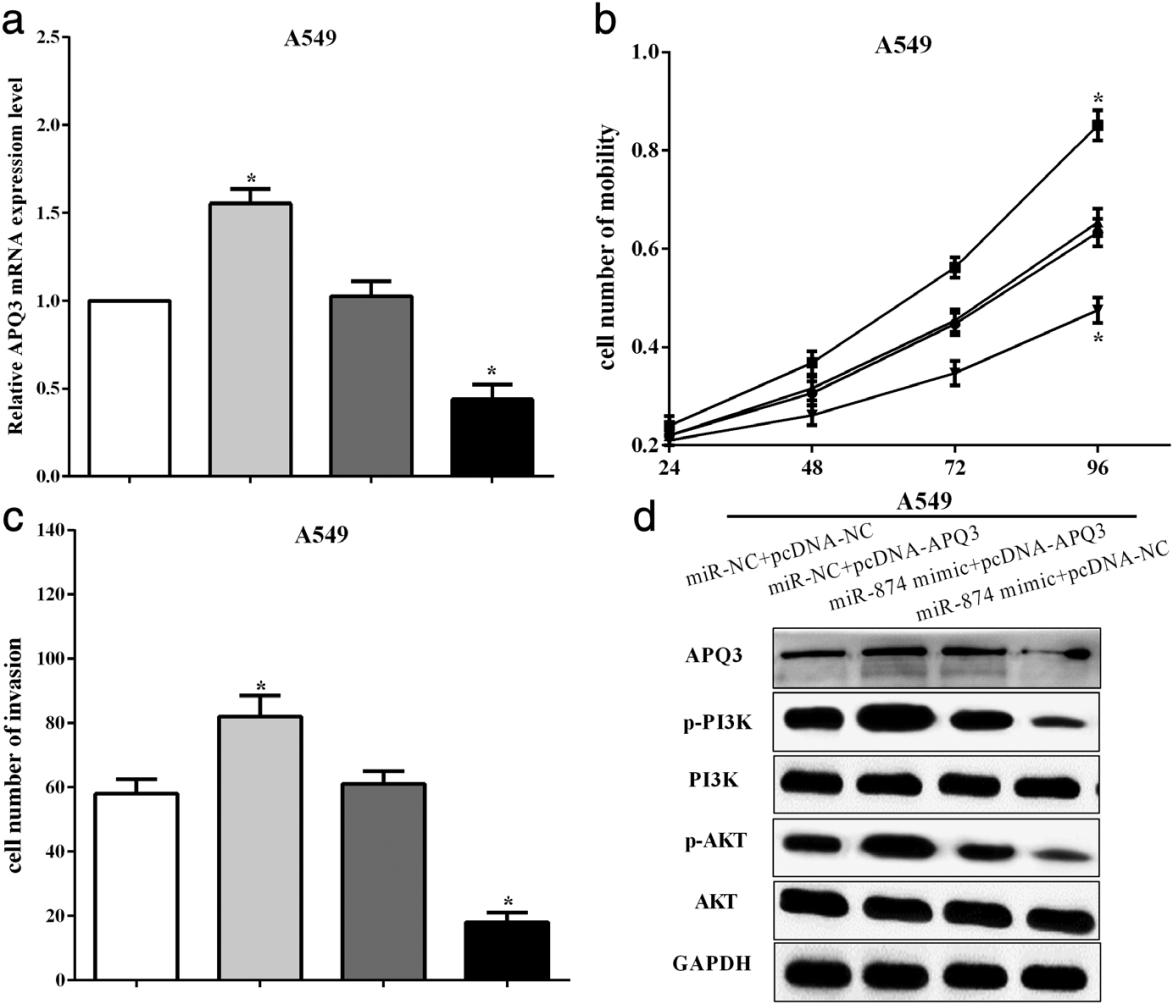
AQP3 reversed partial functions of miR‐874 in A549 cells. (**a**) The transfection efficiency of transfection of pcDNA‐AQP3 or/and miR‐874 mimic in A549 cells. (**b**) Upregulation of APQ3 enhanced cell proliferation and reversed the effect of miR‐874 mimic on cell proliferation. (**c**) Transwell assay revealed that APQ3 reversed partial function of cell mobility in miR‐874 mimic‐transfected cells. (**d**) Upregulation of APQ3 could reverse the suppressive effect of miR‐874 on the expression of p‐PI3K and p‐AKT in A549 cells. (**a**) (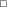) miR‐NC+pcDNA‐NC, (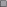) miR‐NC+pcDNA‐AQP3, (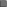), miR‐874 mimic+pcDNA‐AQP3, (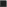) miR‐874 mimic+pcDNA‐NC; (**b**) (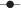) miR‐NC+pcDNA‐NC, (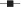) miR‐NC+pcDNA‐AQP3, (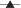), miR‐141 mimic+pcDNA‐AQP3, (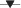) miR‐141 mimic+pcDNA‐NC; (**c**) (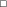) miR‐NC+pcDNA‐NC, (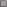) miR‐NC+pcDNA‐AQP3, (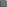), miR‐874 mimic+pcDNA‐AQP3, (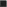) miR‐874 mimic+pcDNA‐NC.

To further elucidate the functional role of the AQP3 gene in miR‐874‐mediated cells, a plasmid expressing AQP3 (pcDNA‐AQP3) was transfected into A549 cells containing miR‐874 mimic, and the transfection efficiency is shown in Fig 5a. CCK‐8 and transwell assays were used to calculate cell proliferation and mobility in A549 cells. As a result, upregulation of AQP3 enhanced cell proliferation (*P* < 0.05) and reversed the effect of miR‐874 mimic on cell proliferation (*P* < 0.05) (Fig 5b). Similar results were found in calculating cell mobility, and transwell assay revealed that AQP3 increased cell mobility in A549 cells (*P* < 0.05), and AQP3 reversed partial function of cell mobility in miR‐874 mimic‐transfected cells (*P* < 0.05) (Fig 5c). In addition, overexpression of AQP3 enhanced the expression of p‐PI3K and p‐AKT. Meanwhile, upregulation of AQP3 could reverse the suppressive effect of miR‐874 on the expression of p‐PI3K and p‐AKT in A549 cells (Fig 5d). All the results revealed that miR‐874 by directly targeting the 3′‐UTR of AQP3 mRNA inhibited EMT and suppressed the phosphorylation of the PI3K/AKT signaling pathway in NSCLC.

### miR‐874 suppressed the xenograft growth in vivo

**Figure 6:**
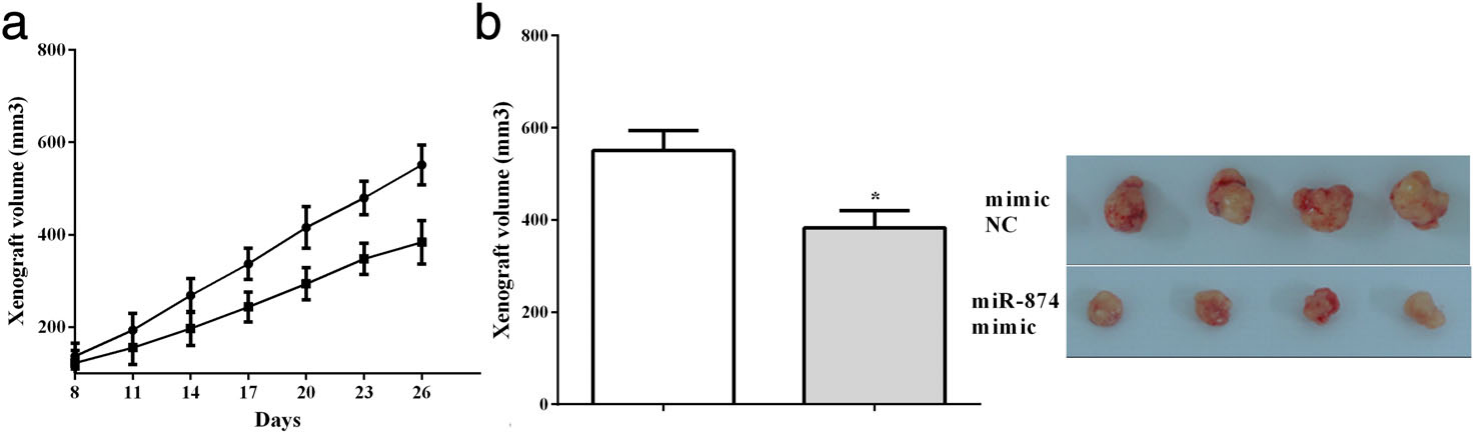
miR‐874 suppressed the xenograft growth in vivo. (**a**) miR‐874 mimic had a slower growth rate than that of the control group. (**b**) The tumor volume of cells that overexpressed miR‐874 was smaller than the tumor volume of the control group. (**a**) 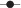 mimic NC, (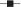) miR‐874 mimic; (**b**) 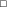 mimic NC, (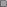) miR‐874 mimic.

A549 cells that stably transfected the miR‐874 mimic or control plasmid were applied to inject subcutaneously into the nude mice. The xenograft tumor volume was calculated every three days and the group that were transfected with the miR‐874 mimic had a slower growth rate than the control group (Fig 6a). We also discovered the tumor volume of miR‐874 overexpressing cells was smaller than that of the control group (*P* < 0.05) (Fig 6b), which indicated that overexpression of miR‐874 inhibited the growth of the xenograft of NSCLC cells.

## Discussion

NSCLC accounts for 85% of lung cancer, usually invading the neighboring tissues and establishing secondary tumors.^21^, ^22^ Therefore, we urgently need to identify new therapeutic targets and molecular mechanisms for the treatment of NSCLC.

miRNAs regulate gene expression and then mediate cell progression through directly binding to the 3′‐UTR of target mRNAs at post‐transcriptional level.^5^, ^23^ miR‐874 acts as a tumor suppressor and suppresses proliferation, migration and invasion in gastric cancer.^24^ Similarly, Leong *et al*. previously reported that in their study miR‐874 inhibited cell growth and colony formation and enhanced apoptosis in hepatocellular carcinoma.^25^ Consistent with all these findings, in our study we discovered that miR‐874 exhibited low expression in NSCLC and downregulation of miR‐874 predicted poor five‐year survival. Overexpression of miR‐874 suppressed the proliferative and invasive capacities in A549 and H1299 cells. Consistent with the findings in gastric cancer,^26^ miR‐874 targeted AQP3 through directly binding to the 3′‐UTR of AQP3 mRNA, and miR‐874 regulated the expression of AQP3 in NSCLC. Here, for the first time, we propose that miR‐874 targets AQP3 in lung cancer and inhibits cellular processes through AQP3.

AQP3, a member of the aquaporin family, is widely distributed in many tissues.^12^, ^13^ AQP3 enhances cell growth and inhibits cell apoptosis in pancreatic cancer.^16^ Similarly, Chen *et al*. found in their study that AQP3 was a sperm water channel and contributed to postcopulatory sperm osmoadaptation and migration.^27^ Our results are consistent with all the above findings, and we discovered that AQP3 was overexpressed in NSCLC tissues and cell lines compared with the noncancer tissues and cell lines. In addition, our results are consistent with the findings in hepatocellular carcinoma,^28^ and we found that miR‐874 suppressed the EMT by downregulating E‐cadherin and upregulating N‐cadherin and Vimentin expression in A549 cells. Moreover, overexpression of AQP3 could partially reverse the roles of miR‐874 on cell proliferation and mobility in A549 cells. The expression of AQP3 was mediated by miR‐874 and inhibited the phosphorylation of PI3K/AKT signaling pathway, which were consistent with the results of Xu *et al*. in gastric cancer.^29^ Previous studies have indicated that phosphorylation of PI3K and AKT blocks the transmission of downstream signals to regulate the proliferation of tumor cells. Our results show that overexpression of AQP3 promotes phosphorylation of PI3K and AKT, indicating that AQP3 can activate the PI3K/AKT pathway. We propose that miR‐874 inhibits the PI3K/AKT pathway in lung cancer. All the findings in this study indicated that miR‐874 regulated the expression of AQP3 and suppressed cell proliferation and mobility in NSCLC. However, the direct evidence that miR‐874 regulated the PI3K/AKT signaling pathway may be through APQ3 is still unclear, and this will be investigated in a future study.

The expression of miR‐874 was lower in NSCLC tissues than the corresponding adjacent nontumor tissues. Downregulation of miR‐874 predicted poor prognosis in NSCLC. Cell proliferation and mobility were suppressed by overexpression of miR‐874, while they were enhanced by knockdown of miR‐874. miR‐874 was found to directly bind to the 3′‐UTR of AQP3 mRNA and mediated the expression of AQP3 in NSCLC cells. What is more, miR‐874 inhibited the proliferation and mobility by directly targeting AQP3 in NSCLC cells. miR‐874 inhibited the EMT and suppressed the phosphorylation of PI3K/AKT in NSCLC. AQP3 partially reversed the suppressive roles of miR‐874 in A549 cells. In addition, miR‐874 inhibited the growth of NSCLC cells in vivo.

## Disclosure

The authors declare no conflict of interest.
